# Spinal anesthesia in the percutaneous fixation of fragility fractures of the pelvis

**DOI:** 10.1016/j.tcr.2022.100735

**Published:** 2022-11-23

**Authors:** A.H.M. Mennen, R.W. Peters, M.V.H. Rutten, D. van Embden

**Affiliations:** a Amsterdam UMC location University of Amsterdam, Department of Surgery, Meibergdreef 9, Amsterdam, the Netherlands; b Amsterdam UMC location University of Amsterdam, Department of Anesthesiology, Meibergdreef 9, Amsterdam, the Netherlands

**Keywords:** Spinal anesthesia, Pelvic fragility fracture, Osteoporotic fracture, Percutaneous fixation, Case reports

## Abstract

**Introduction:**

The number of geriatric patients with a pelvic ring fracture is rising and minimal invasive fixation techniques are increasingly popular. The patient characteristics of these fragile patients are similar to those of patients with a proximal femur fracture. In the field of proximal femur fracture surgery spinal anesthesia is a very commonly used anesthetic technique in this more fragile patient population.

**Methods:**

All patients were treated between January 2022 and May 2022 in the Amsterdam UMC location AMC in The Netherlands. The operations were performed by a surgeon who specialized in pelvic and acetabular fracture surgery in a hybrid operating theatre. All patient in this case series received spinal anesthesia using 2–2.5 ml glucosated bupivacaine 5 mg/ml.

**Results:**

We describe, for the first time, four cases of percutaneous pelvic ring fracture fixation using spinal anesthesia. There were no perioperative or direct postoperative complications. Patients quickly regained the ability to mobilize, reported little pain complaints, and could be safely discharged to either a rehabilitation center or home.

**Conclusion:**

We believe spinal anesthesia could be a safe alternative to general anesthesia for the percutaneous fixation of pelvic ring injuries in a selected group of frail elderly patients. A proper assessment should determine whether or not spinal anesthesia is an option in pelvic fracture fixation, taking patient preference, the advice of the anesthetist, the choice of operative technique, and fracture pattern into consideration.

## Introduction

While studies in other high-income countries show a decrease in the incidence of hip fractures, the number of patients with a pelvic fracture increased by 25 % in the last 5 years [1]. When taking incidence rates of the last decades into consideration, an astonishing increase of almost 400 % was reported [2]. Historically, pelvic ring fracture fixations were open procedures associated with significant morbidity such as severe blood loss. However, more recently percutaneous techniques such as screw fixation of the pubic rami and sacral fractures or the IN-FIX technique have grown in popularity as a safe and reliable treatment option for elderly patients with a pelvic fragility fracture. This is mostly due to the fact that this technique is associated with less surgery-related morbidity and shorter operating time [3].

In hip fracture and joint replacement surgery, spinal anesthesia has been part of standard care for decades. Spinal anesthesia has been incorporated in treatment protocols for Total Hip Replacement [4], [5]. Whether or not spinal anesthesia for the more fragile geriatric population such as hip fracture patients is superior to general anesthesia is still subject of debate. Recent studies have failed to show differences in mortality or postoperative ambulatory rates but a different set of complications come with both of the techniques [6], [7], [8]. For example, it has been described that spinal anesthesia for hip fractures patients is associated with higher rates of urinary tract infections and a longer hospital stay, and general anesthesia could be associated with a higher rate of thrombo-embolic events [6].

As for pelvic fracture fixation, no cases have previously been described in literature of spinal anesthesia in percutaneous pelvic fracture fixation. To demonstrate our experiences, we present four cases of patients who sustained a fragility fracture of the pelvis and underwent percutaneous pelvic fixation under spinal anesthesia.

## Methods

All patients were consecutive cases treated between January 2022 and May 2022 in the Amsterdam UMC location AMC in The Netherlands. The Amsterdam UMC is a level one trauma center and a regional referral hospital for pelvic and acetabular fracture surgery. All patients were operated by a surgeon who specialized in pelvic and acetabular fracture surgery. All patients were operated on in a hybrid operating theatre in which a perioperative CT-scan is routinely performed to check correct K-wire placement for our 7.3 cannulated screw placement. Operating times presented below are including the CT-scanning time, so times are partly dependent on the assisting radiographers' experience levels. All patient in this case series received spinal anesthesia using 2–2.5 ml glucosated bupivacaine 5 mg/ml. The addition of fentanyl was used in some cases, but not in all. The average time it took the anesthesiologist to perform the neuraxial anesthesia was 15 min (range 9–25 min). Although urinary decompression is not mandatory in patients receiving spinal anesthesia, in our institute we do routinely use catheterization in all patients undergoing pelvic surgery.

Two patients used carbasalate calcium and one patient used rivaroxaban prior to surgery. The anticoagulant protocol implemented in our hospital was consulted to determine if the patients should stop their medication prior to the neuraxial block. Thrombocyte aggregation inhibitor treatment can continue per usual. It is common practice to interrupt vitamin K-antagonist treatment (VKA). If a patient is taking VKA the day before the neuraxial block and/or surgery, the INR will be monitored and corrected to <1.8 if necessary. The advised interval between the last dosage of direct oral anticoagulantia (DOAC) and the placement of a neuraxial block ranges between 2 and 4 days depending on the kidney function of the patient. Low molecular heparin (LMWH) should not be given in the 10 h prior to the neuraxial block, and can be continued at least 4 h after the placement of the neuraxial block.

### Case 1

**Fig. 1:**
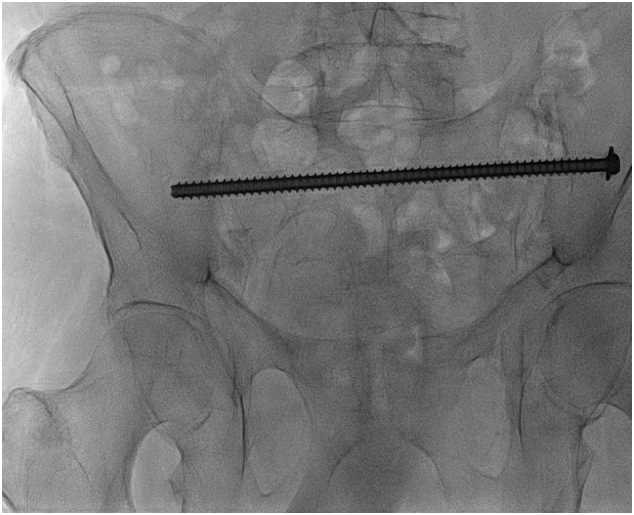
Case 1 with one Trans-Ilium-Trans-Sacral (TITS) screw in S1.

A 76 year old man presented 5 days after a fall at home at the emergency department with severe thoracic trauma. His previous history showed severe alcohol abuse and paralysis of the right hemidiaphragm. He was previously ambulant and lived independently at home. At the emergency department he showed signs of respiratory distress, and imaging showed fractures of ribs 2 to 10 and a large hemopneumothorax on the left. Conservative management focusing on pain relief and oxygen suppletion was started, but after 4 days his clinical status did not improve. He underwent rib fixation and a Video Assisted Thoracoscopy (VATS) was performed to evacuate a hemothorax. From the first day of his admission onwards he developed a delirium. After his pulmonary status and delirium improved, around five weeks after the initial trauma, the patient was still unable to mobilize and complained of severe sacral pain. The CT showed a sacral and pubic ramus fracture, both located on the left side. The fracture was classified as a type 3c according to Rommens. Using shared decision making it was decided to operatively stabilize the sacral fracture for pain relief and in order to optimize his mobilization. The patient was anxious for general anesthesia due to his recent delirium. Despite the lack of evidence that patients undergoing spinal anesthesia have a lower risk of delirium, after shared decision making the patient opted for spinal anesthesia. During surgery one Trans-Ilium-Trans-Sacral (TITS) screw in S1, from left to right, was placed (Fig. 1. The total operation time was 41 min including a perioperative CT-scan to check the K-wire placement. The direct postoperative course was uncomplicated and the patients' ability to walk improved quickly. He was able to mobilize well with a walking aid and could be discharged 12 days postoperatively to a rehabilitation center.

### Case 2

**Fig. 2:**
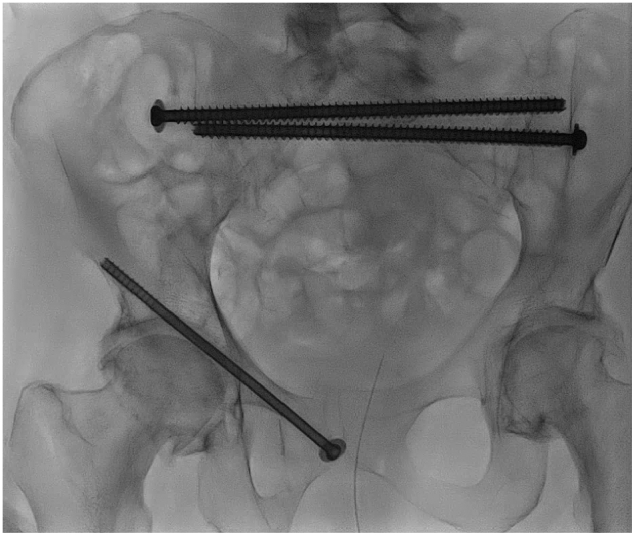
Case 2 with bilateral TITS sacral screws in S1 and a right sided ramus screw.

A 79 year-old female patient was presented at our emergency department. Her medical history included COPD Gold IV, frequent falls and multiple episodes of acute myocardial infarction. She lived independently at home and became injured after a low energy fall. Her pelvic radiograph showed a right sided pubic ramus fracture. Two weeks after her initial presentation her situation deteriorated in terms of a severe increase of pain. She was unable to mobilize and was admitted to a nursing home. Additional CT-scan showed concomitant bilateral displaced sacral fractures and the fracture was classified as a fragility fracture type 4c according to Rommens [10]. In accordance with the patient's wishes it was decided to stabilize her severe fragility fracture of the pelvis for pain relief and to improve her mobility. Preoperative analysis showed severe pulmonary emphysema, including oxygen saturations of around 77 % without oxygen suppletion without the patient showing signs of respiratory distress. Because of her severe lung disease, general anesthesia was deemed unsafe and our anesthetist decided together with our pulmonologist that spinal anesthesia was deemed the most safe option for her to undergo screw fixation. We used bilateral TITS sacral screws in S1 and a right sided ramus screw. Perioperative CT scan was performed and showed correct placement of the screws (see Fig. 2). The total operating time was 44 min. The direct postoperative course was uncomplicated and the patients mobility and pain improved rapidly. She was able to walk a couple of steps in the hospital with a walking aid and could be discharged to a rehabilitation center after 7 days of admission.

### Case 3

**Fig. 3:**
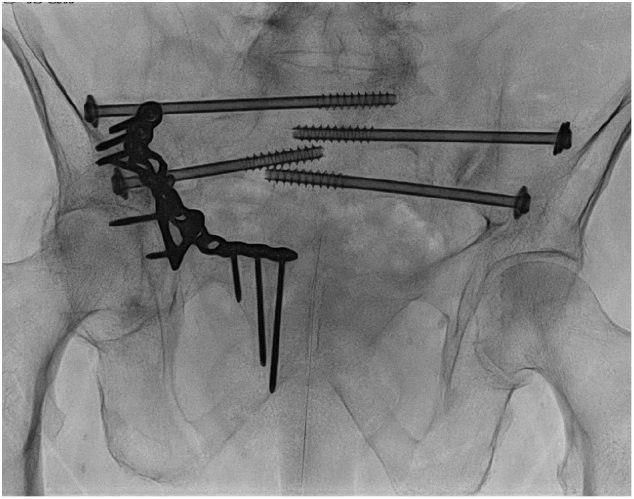
Case 3 with four SI screws in S1 an S2 bilaterally, and previously placed acetabular plate fixation.

A 83 year old man was presented at our Emergency Department three weeks after a fall at home. He was first presented at a local hospital with a severe aspiration-pneumonia, bilateral sacral fractures, and pubic ramus fracture on the right. After he recovered from his pneumonia he was admitted to a nursing home where he was unable to mobilize. He became bedridden and had an increasing need for opioids to manage his pain. He was referred to our hospital by the nursing home physician. A new CT-scan was performed and it showed displaced bilateral U-shaped sacral fractures (Rommens type 4b), L5 transverse process fractures, and a healing ramus fracture in a previously surgically stabilized acetabular fracture on the right. His other relevant comorbidities include; generalized weakness, electrolyte disturbances, severe peripheral arterial disease, diabetes mellitus, alcohol abuse, and an acute myocardial infarction. Because the patient was previously living independently at home and his strong wish was to recover and to return to his home, we performed percutaneous screw fixation under spinal anesthesia. The total operation time was 58 min, in which we placed four SI screws in S1 an S2 bilaterally. A perioperative CT showed correct placement of the screws (Fig. 3). The direct postoperative course was uncomplicated and the patient was able to mobilize with help of the physiotherapist. The patient was discharge to a rehabilitation center after 8 days of hospital admission.

### Case 4

**Fig. 4:**
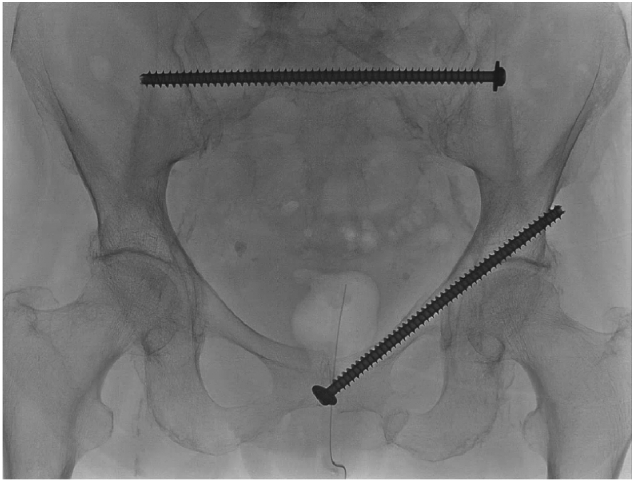
Case 4 with one TITS S1 sacral screw and one pubic ramus screw.

A 84 year old female was referred to our hospital after a fall at home two weeks prior. The patient sustained a bilateral sacral fracture with some displacement on the left side, and pubic rami fractures on the right (Rommens type 4c). She was unable to walk due to an increasing amount of pain. She had and extensive medical history including a cT1aN0M0 lung nodule in the left upper lobe which was treated with radiotherapy, pT2N1(sn) right sided breast cancer treated with ablation and radiotherapy of the chest wall and the axilla and hormone treatment. She suffered from shortness of breath as a result to the previous radiotherapy, COPD, chronic heart failure and atrium fibrillation. A couple of weeks prior to her fall she received a pacemaker because of severe pulmonal hypertension. She was previously mobile, walked without walking aids and lived independently at home. Because of her respiratory problems, spinal anesthesia was the preferred method of anesthesia by the patient, anesthesiologist and the surgeon. We placed a TITS S1 sacral screw and a pubic ramus screw. A CT-scan was performed to check the K-wire positioning, and because of a slightly anterior position of the sacral screw, the screw was replaced (Fig. 4). The total operating time was 75 min, including replacement of the screw. The patient recovered very well and was able to walk pain free with walking aids soon after surgery. She was discharged home after a hospital admission of 6 days.

### Outcome and follow-up

After a follow-up period of four to six months, the patients described in case 1 and 2 were doing well and reported no pain, mobilized using a walker, and were able to live independently at home. The patient of case 3 experienced no pain and was able to walk short distances with the use of a walker at a follow-up period of 4 months, but decided to start palliative care due to other complaints. The patient of case 4 was last seen at the out-patient clinic six weeks post-surgery and at this point was doing well without any complaints. She had returned to her previous place of residence and was able to mobilize with the use of a walker. Three months post-surgery she passed away from unrelated causes.

## Discussion

This case series demonstrates that the patient characteristics of elderly patients with a pelvic fragility fracture often resemble those with hip fractures: high rates of comorbidities and an increased risk of complications related to immobilization. Both pelvic and hip fractures in elderly occur after low energy trauma, such as fall from standing height. Despite its similarities, elderly patients with pelvic fractures are currently not treated according to the same treatment principles as hip fracture patients. Early fixation and full weight bearing in hip fracture patients in order to reduce complications is generally adopted and common practice all over the world. Remarkably, this is not the case in patients with a fragility fracture of the pelvis. Despite the fact that these fractures are fairly common, there is no consensus on best treatment strategy and this lack of an evidence-based treatment strategy results in a wide variety of clinical practice. In recent literature several authors have proposed treatment strategies mimicking the treatment of hip fracture patients in whom early fixation followed by immediate weightbearing is standard care [11], [12].

In hip fracture surgery spinal anesthesia is very common and incorporated in most treatment guidelines. There has been an increased use of spinal anesthesia of 50 % between 2007 and 2017 [13]. Despite the increased use of spinal anesthesia, no advantage of spinal anesthesia was ever proven over general anesthesia in hip fracture patients [6], [7], [8]. In a recent large randomized controlled trial no difference between techniques in terms of mortality and walking ability was shown for the hip fracture patients [7]. Despite the recent randomized controlled trial, Van Waesberge et al. state, a lot of important questions remain unanswered, such as whether or not general anesthesia increases the risk for myocardial infarction [14]. Therefore, in selected patients anesthetist do still prefer spinal anesthesia over general anesthesia as both techniques come with different challenges in this frail patient category. Variations to a spinal anesthesia could be used in cases that have a risk of a longer operating time, spinal anesthesia with glucosated bupivacaine last for about an hour and a half. In cases that have the risk of running longer a spinal catheter an epidural catheter or a combined spinal epidural technique could be feasible.

Contra-indication for using spinal anesthesia is the presence of aortic stenosis. Therefore a proper assessment should be done prior to accepting these patients for spinal anesthesia. Whether or not spinal anesthesia is an option in pelvic fracture fixation is also very dependent on the choice of operative technique and, of course, fracture pattern. A number of studies indicate early diagnosis by performing an early CT-scan and percutaneous techniques have been increasingly popular in order to regain immediate weightbearing mobility in these elderly [3], [15]. Some surgeons prefer sacral bars or spinal pelvic fixation for fragility fractures of the sacrum [16], [17]. In cases like these, general anesthesia will be preferable, because of the prone positioning. One could debate, depending on the patients' comorbidities whether TITS or SI screws is perhaps preferable in the more fragile patients. Also, if a surgeon decides in favor of, for example, anterior plating of a pubic rami fractures or the symphysis, in which muscle relaxation is preferable or some blood loss is anticipated, general anesthesia is preferable [17]. Again, one should assess and discuss the patient and decide together with the anesthetist whether or not spinal anesthesia is an option in every single case. As this case series shows, even in cases of the *very* fragile patients, some could still benefit from percutaneous techniques such as screws or IN-FIX. Finally, the patient should be asked about its preferences and should be well informed about the options and potential complications of both anesthetic techniques.

## Conclusion

In this study the use of spinal anesthesia for percutaneous fixation of fragility fractures of the pelvis has been described for the first time. We believe spinal anesthesia could be a safe alternative to general anesthesia in a selected group of patients for the percutaneous fixation of pelvic ring injuries in elderly. We advise careful and shared decision making with both patient and anesthesiologist, and thorough preoperative planning to asses if the fracture pattern is admissible for a percutaneous fixation technique. Future randomized controlled trials should determine which patients benefit the most from spinal anesthesia compared to general anesthesia.

## Funding

No funding was received for conducting this study.

## Ethical approval

The study was approved by the local ethics committee.

## Informed consent

Informed consent was acquired of all patients included in the case series.

## Conflict of interest

The authors state that there are no interests to disclose.
